# Serine 204 phosphorylation and O-*β*-GlcNAC interplay of IGFBP-6 as therapeutic indicator to regulate IGF-II functions in viral mediated hepatocellular carcinoma

**DOI:** 10.1186/1743-422X-8-208

**Published:** 2011-05-08

**Authors:** Waqar Ahmad, Khadija Shabbiri, Bushra Ijaz, Sultan Asad, Noreen Nazar, Shazia Nazar, Kiran Fouzia, Humera Kausar, Sana Gull, Muhammad T Sarwar, Imaran Shahid, Sajida Hassan

**Affiliations:** 1 Applied and Functional Genomics Lab, Centre of Excellence in Molecular Biology, University of the Punjab, Lahore-53700, Pakistan; 2 Department of Chemistry, GC University Lahore, Pakistan

## Abstract

Hepatocellular carcinoma is mainly associated with viral hepatitis B and C. Activation of cell growth stimulator IGF-II gene is observed in tumor formation especially in viral associated hepatocellular carcinoma. Elevated IGF-II levels are indicator of increased risk for cholangiocellular and hepatocellular carcinomas through over saturation of IGF-II binding capacities with IGF receptors leading to cellular dedifferentiation. In HCV, core protein is believed to trans-activate host IGF-II receptor through PKC pathway and the inhibition of tumor cell growth can be achieved by blocking IGF-II pathway either at transcriptional level or increasing its binding with IGFBPs (Insulin like growth factor proteins) at C-terminal, so that it is not available in free form. IGFBP-6 is a specific inhibitor of IGF-II actions. Affinity of IGFBPs with IGFs is controlled by post-translational modifications. Phosphorylation of IGFBPs inhibits IGFs action on target cells while O-glycosylation prevents binding of IGFBP-6 to glycosaminoglycans and cell membranes and resulting in a 10-fold higher affinity for IGF-II. *O*-glycosylation and phosphorylation operate the functional expression of cellular proteins, this switching on and off the protein expression is difficult to monitor in vivo. By using neural network based prediction methods, we propose that alternate O-*β*-GlcNAc modification and phosphorylation on Ser 204 control the binding of IGFBP-6 with IGF-II. This information may be used for developing new therapies by regulating IGFBP-6 assembly with IGF-II to minimize the risk of viral associated hepatocellular carcinoma. We can conclude that during HCV/HBV infection, *O*-*β*-GlcNAc of IGFBP-6 at Ser 204 diminish their binding with IGF-II, increase IGF-II cellular expression and promote cancer progression which can lead to hepatocellular carcinoma. Furthermore, this site can be used for developing new therapies to control the IGF-II actions during viral infection to minimize the risk of hepatocellular carcinoma.

## Introduction

Among the highly malignant human tumors, hepatocellular carcinoma is the most common. It is the fifth most prominent tumor in the world and is the third most widespread cause of cancer-related death [1]. HCC progression is a multi-step process along with multiple factors based etiology. Major risk factors contributing to HCC include HCV and HBV infection along with alcohol intake and metastasis of cancer in other parts of the body like colon [2]. The malignant transformation process is influenced by a number of growth factors, receptors and other related proteins. Among these related proteins, IGF axis is one of the very important disease contributors [3]. The insulin-like growth factor (IGF) system regulates growth, development and function of glands through complex interactions with other growth factors and hormones [4]. IGF has structure similarities to insulin [5]. IGF system is composed of two ligands (polypeptide growth factors, IGF-I and IGF-II), two receptors (IGF-IR and IGF-IIR), and six high-affinity binding proteins (IGFBPs) [6]. IGFs bind to a number of proteins including insulin receptors (IGF-I receptor and IGF-II receptor) and serum carrier proteins. IGFs exert their biological actions by interacting with specific receptors localized on the cell membrane [7,8].

IGF-I binds with high affinity to IGF-I receptor whereas IGF-II binds with IGF-II receptor more effectively [9]. IGF-II responses are mediated by G-proteins singling. IGF-II receptor stimulates cellular responses like proliferation and motility when interact with IGF-II [10]. IGF-II has cell replication promoting effects and is also termed as multiplication stimulating activity (MSA) [11]. IGF-II is closely related to insulin like fetal growth peptide produced by liver. In a variety of neoplasms IGF-II is reported to be highly over expressed [12]. Furthermore aberrant IGF-II expression is also thought to be involved in liver carcinogenesis [13]. High concentration of IGF-II is also been found in the cancerous liver cell lines i.e. Huh-7 and HepG2. While IGF-II transgenic mice showed increased risk of HCC [14,15]. Recently IGF-II has been proposed as serum marker of human HCC [16]. In case of hepatocarcinogenesis, increased expression of IGF-II, protease activity of IGF-binding proteins and IGF-I receptor along with down regulation of IGF-II receptor is considered to play an important role in the disease progression [14]. In HCV-related cirrhosis patients there is significant increase in the IGF-II expression which clearly indicates its strong link with HCV [7]. In HCV chronic hepatitis the continuous process of hepatocytes damage and regeneration can possibly inflame uncontrolled growth of hepatocytes leading to malignant transformations possibly due to aberrant growth regulation or mitogenic factors disruption [17]. The exact mechanism of correlation of HCV and IGF-II deregulation is still not fully understood.

IGFBP-6 an important member of IGFBPs family is a relatively specific inhibitor of IGF-II actions. IGFBP has the highest affinity among IGFBPs to bind with IGF-II along with 20 to 100 fold preference of binding to IGF-II rather than IGF-I. Non proliferative state is usually related with IGFBP-6 expression. IGFBP-6 expression is generally stimulated by different agents like retinoic acid [18]. It consists of three domains of equal size. The N and C-terminals are internally sulfide linked and share a high degree of sequence homology across the IGFBPs family [18,19]. The C-domain of IGFBP-6 reacts with IGF-II at thyroglubulin type 1 fold [20]. The IGFII binds itself on IGFBP-6 hydrophobic end located between α-helix and the first and second loop of the first strand. Many amino acid residues on the surface like, Val178, Ser203, Ser 204, Gly206, Ala182 and Pro188 are also considered as the binding supporter of the IGFII. This is still need to be confirmed experimentally [21].

Cell systems in which IGFBP-6 has been shown to inhibit IGF-II induced effects such as proliferation, differentiation, cell adhesion, and colony formation include osteoblasts, keratinocytes, myoblasts, and colon cancer cells [22]. Post-translational modifications (PTM) regulate the affinity of IGFBPs for IGFs and this is the principal mechanism involved in regulating IGF bioavailability during folliculogenesis [23]. IGFBP-6 undergoes a number of posttranslational modifications, including proteolytic cleavage, phosphorylation and glycosylation [24]. Phosphorylation of IGFBP-6 leads to change its affinity with IGFs and it inhibits IGF-II action on target cells [7,22]. Glycosylation is also very much known PTM in IGFBPs especially in IGFBP-6 in human, rat and mouse [19,20,24]. *O*-glycosylation prevents binding of IGFBP-6 to glycosaminoglycans and cell membranes and resulting in a 10-fold higher affinity for IGF-II. *O*-glycosylation also delays and resists the proteolysis of IGFBP by chymotrypsin and trypsin than non-glycosylated IGFBP-6 [19,20].

It has been documented but not extensively studied that high levels of IGF-II in hepatocellular carcinoma can be control by parental imprinting or changes in methylation pattern in IGF-II genes [25]. In this study we describe another phenomenon that may regulate IGF-II functions through post-translational modifications in IGFBP-6. We describe potential phosphorylation and O-*β*-GlcNAc sites, and their possible interplay in IGFBP-6 leading to change affinity with IGF-II and modify the functions of IGF-II, which have been predicted and analyze using different prediction methods available. On the basis of potential phosphorylation and glycosylation interplay in conserved residues of IGFBP-6, the possible roles played by these post-translational modifications in regulating the functions of IGFBP-6 are analyzed.

## Materials and methods

The sequence data used to predict potential phosphorylation and glycosylation sites of IGFBP-6 of *Homo sapiens *was retrieved from the SWISS-PROT sequence database [26]. The entry name was IBP-6 Human with the primary accession number P24592. BLAST search was made using NCBI database which finds regions of local similarity among the sequences of proteins or nucleotides, and can be used to elucidate evolutionary relationships [27]. The search was performed on known species of different mammals. Six IGFBP-6 sequences with highest bit score values were selected as given in Table 1. The mammals selected were Homo *sapiens *(Human, P24592), *Mus musculus *(Mouse, P47880), *Ovis aries *(Sheep, B5AN56), *Rattus norvegicus *(Rat, P35572), *Sus sacrofa *(Pig, A9NJ32) and *Bos taurus *(Bovin, Q05718). All the six sequences were multiple aligned using ClustalW [28]. ClustalW is a general purpose multiple sequence alignment program for DNA or protein.

**Table 1:** Different IGFBP-6 proteins used for multiple alignment

Species name	**Accession no**.	Identity	Score	E-Value
Human (*Homo sapiens*)	P24592	100%	1,322	1.0 × 10^-144^

Pig (*Sus scrofa*)	A9NJ32	84%	1,101	1.0 × 10^-118^

Bovin (*Bos taurus*)	Q05718	83%	1,079	1.0 × 10^-116^

Sheep (*Ovis aries*)	B5AN56	82%	1,054	1.0 × 10^-113^

Mouse (*Mus musculus*)	P47880	70%	880	1.0 × 10^-92^

Rat (*Rattus norvegicus*)	P35572	66%	783	2.0 × 10^-81^


The IGFBP-6 sequence used in this study was 

 "MTPHRLLPPLLLLLALLLAASPGGALARCPGCGQGVQAGCPGGCVEEEDGGSPAEGCAEAEGCLRREGQECGVYTPNCAPGLQCHPPKDDEAPLRALLLGRGRCLPARAPAVAEENPKESKPQ

AGTARPQDVNRRDQQRNPGTSTTPSQPNSAGVQDTEMGPCRRHLDSVLQQLQTEVYRGAQTLYVPNCDHRGFYRKRQCRSSQGQRRGPCWCVDRMGKSLPGSPDGNGSSSCPTGSSG".

### Prediction of post-translational modifications

#### Prediction of phosphorylation residues and related kinases

Phosphorylation potential for human IGFBP-6 was predicted by using NetPhos 2.0 (http://www.cbs.dtu.dk/services/NetPhos/) server [29]. This is a neural network-based program that predicts the potential phosphorylation sites for each Thr, Ser and Tyr residues. The minimum threshold value used to predict phosphorylation is 0.5. Disphos 1.3 (http://www.ist.temple.edu/disphos/) server [30] was also used for the prediction of possible phosphorylation sites in human IGFBP-6.

Kinase specific phosphorylation sites in human IGFBP-6 were predicted by NetPhosK 1.0 server (http://cbs.dtu.dk/services/NetPhosK) [31]. The NetPhosK 1.0 predicts the kinase specific acceptor substrates including Ser, Thr and Tyr. KinasePhos 2.0 (http://kinasephos.mbc.nctu.edu.tw/case2.html) server [32] was also used for kinase prediction.

For the purpose of evaluating experimentally verified phosphorylation sites on human IGFBP-6, Phospho.ELM database (http://phospho.elm.eu.org) was used [33]. This database contains a collection of experimentally confirmed Ser, Thr and Tyr residues in eukaryotic proteins.

#### Prediction of o-glycosylated residues and YinOYang sites

*O-β*-GlcNAc modification potential sites can be predicted by YinOYang 1.2 (http://www.cbs.dtu.dk/services/YinOYang/). This program can predict the potential phosphorylation sites as well and hence predicting the Yin-Yang sites with highly uneven threshold that is adjusted in accordance with amino acid surface accessibility [34-37]. We also used OGPET (http://ogpet.utep.edu/OGPET/) [38] for predicting *O*-glycosylation at Ser and Thr residues.

#### Protein structure analysis

As there was no template model of IGFBP-6 available in protein data bank [39], we designed an ab-initio model by using software I-TASSER (http://zhanglab.ccmb.med.umich.edu/I-TASSER/) [40]. Data in sequence form was uploaded to the server. Model with high C-score was selected as ab-initio model. To view and analyze 3D structure Jmol (http://jmol.sourceforge.net/) [41] and PYmol (http://www.pymol.org/export) [42] programs were used. To assess, whether the predicted Ser and Thr residues have surface accessibility for post-translational modifications, NetSurfP (http://www.cbs.dtu.dk/services/NetSurfP/) was used [43]. We also used Scansite server (http://scansite.mit.edu/motifscan_seq.phtml) to check surface ability of IGFBP-6 to solvents and for PTMs [44].

#### Neural networks-based prediction methods

Above described prediction methods are artificial neural network-based and have been extensively used in predicting the potentials of proteins for post-translational modifications and in biological sequence analysis [45]. These methods are designed by memorizing the known sequence environment data of glycosylated/phosphorylated Ser/Thr and non-glycosylated/non-phosphorylated Ser/Thr sites. The results obtained from all the networks are sigmoidally arranged and averaged to obtain a value between zero and one by these prediction methods. Usually a threshold of 0.5 is used for prediction, which means that a site with an output of more than 0.5 is assigned as having a potential to be glycosylated or phosphorylated. NetPhos 2.0 predicts phosphorylation on the OH- function of Ser, Thr or Tyr residues with a sensitivity range of 69-96%. YinOYang 1.2 employed the sequence data to train a jury of neural networks on 40 experimentally determined O-GlcNAc acceptor sites for recognizing the sequence context and surface accessibility. This method is efficient in a cross validation test as it correctly identifies 72.5% of the glycosylated sites and 79.6% of the non-glycosylated sites in the test set, verifying the Matthews correlation coefficient of 0.22 on the original data, and 0.84 on the augmented data set. This method has the capability to predict the YinOYang sites that can be glycosylated and alternatively phosphorylated on Ser/Thr or Tyr residues.

## Results

### Alignment of sequences for the determination of conserved status of Ser/Thr residues within IGFBP-6

**Figure 1:**
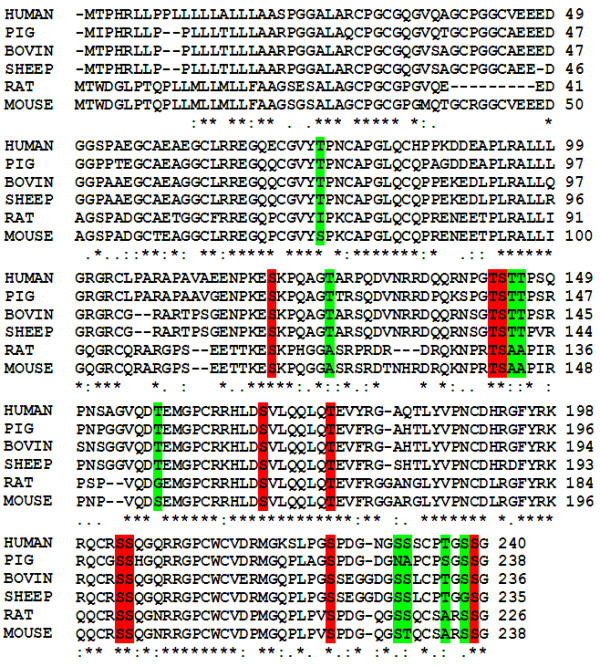
**Multiple alignments of six vertebrates sequences (Human, Bovin, Sheep, Pig, Mouse and Rat)**. These different sequences were ordered as aligned results from ClustalW. The consensus sequence is marked by an asterisk, conserved substitution by a double dot, and semi conserved substitution by a single dot.

To determine conserved and conserved substituted Ser and Thr residues within each subtype, human IGFBP-6 protein FASTA sequence was aligned with other mammals (Figure 1). It is clear from the figure that Ser 120, 144, 169, 203, 204, 225 and 239; and Thr 143 and 176 were highly conserved in mammals. Meanwhile, Ser 231 and 232; and Thr 75, 126, 145, 146 and 236 showed conserved substitutions within mammals.

### Acquiring experimentally verified S/T/Y residues

Data for experimentally confirmed S/T/Y residues was searched from Phospho.ELM (http://phospho.elm.eu.org) and UniprotKB (http://www.uniprot.org). Although all IGFBP sub forms are subjected to be phosphorylated, however, there is no experimentally verified phosphorylation site.

### Prediction of Phosphorylation Sites

**Figure 2:**
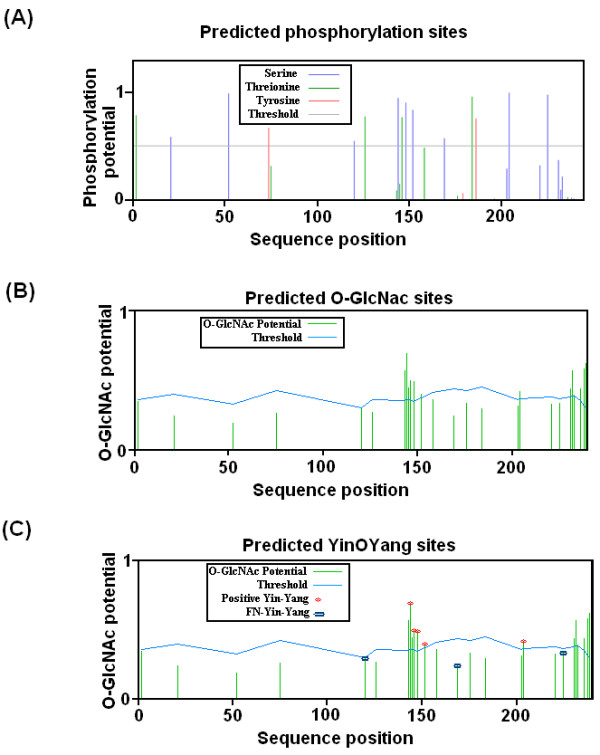
**Graphic representation of the potential Ser, Thr, and Tyr residues for phosphorylation and o-glycosylation modification at human IGFBP-6**. **A) **Predicted potential sites for phosphate modification on Ser and Thr residues. The light gray horizontal line indicates the threshold for modification potential. The blue, green and red vertical lines show the potential phosphorylated Ser, Thr and Tyr residues, respectively. **B) **Predicted potential sites for o-glycosylation modification of Ser and Thr. *O*-*β*-GlcNAc modification potential of Ser/Thr residues is shown by green vertical line, while the light blue wavy line indicates the threshold for modification potential. **C) **The Yin Yang sites that were positively predicted are shown with red asterisk at the top, while the NP-Yin-Yang sites are shown with purple asterisk on the top of vertical lines. The green vertical lines show the *O*-*β*-GlcNAc potential of Ser/Thr residue and the light blue horizontal wavy line indicates the threshold for modification potential.

Prediction results by NetPhos 2.0 for possible phosphorylation sites revealed that IGFBP-6 possesses high potential for phosphate modification like other subtypes. 15 phosphorylation sites at Ser, Thr and Tyr residues were predicted. In IGFBP-6 there are 9 Ser, 4 Thr, and 2 Tyr residues that are subjected to phosphorylation as shown in Figure 2.

### Prediction of Kinases involved in Phosphorylation

Different kinases are involved in phosphorylation of mammalian IGFBPs so specific phosphorylation substrate potential was assessed by using NetPhosK 1.0. The results obtained from NetPhosK 1.0 had shown the involvement of different kinases in phosphorylation of predicted human IGFBP-6. PKC can phosphorylate Ser (120, 169, 204 and 221) and Thr (126 and 176). Cdc2 can phosphorylate Ser (232, 233, and 239) and Thr (126 and 176). Similarly Cdk5 can phosphorylate Ser (225) and Thr (2, 85 and 146). CK II can phosphorylate Ser 52, PKA phosphorylate serine 120, DNAPK and ATM both can phosphorylate serine 144 and 204. EGFR can phosphorylate Tyr 179 and 196 while GSK3 can phosphorylate Ser 221 and 225 respectively.

The other neural network based program Kinasephos was also used to assess the possible kinases on IGFBP-6. It also predicts many kinases that may be involved in IGFBP-6 protein phosphorylation as given in Table 2.

**Table 2:** Predicted phosphorylation and O-glycosylation sites on IGFBP-6 protein

Substrate	Position	Phosphorylation prediction		Kinase prediction		O-glycosylation prediction		Surface accessibility	
		
		Netphos	Disphos	NetphosK	Kinasephos	YinOYang	OGPET	Scansite	NetSurfP
Thr	2	Y	Y	MAPK, CDK5	-	N	LP	-	E

Ser	21	Y	N	MAPK, CDC2, GSK3	CAM2, CDC2, MAPK, CDK	N	LP	-	B

Ser	52	Y	Y	CK2	CDC2, ATM, IKK	N	VHP	<1	E

Thr	75	P	Y	MAPK, CDK5	CDK	N	VHP	<1	E

Ser	120	Y	Y	PKC, PKA	PKG, CKI	N	LP	-	E

Thr	126	Y	Y	PKC, CDC2	PKC	N	VHP	-	E

Thr	143	N	N	-	MDD	Y	VHP	-	E

Ser	144	Y	Y	-	PKC, CDC2	Y	HP	>1	E

Thr	145	P	Y	PKC	CDK, MDD	Y	HP	>1	E

Thr	146	Y	Y	CDK5	CDC2, CDK	Y	HP	>1	E

Ser	148	Y	Y	DNAPK, ATM	PKA, CKI, ATM	Y	HP	>1	E

Ser	152	Y	N	-	CDC2, IKK	Y	VHP	-	E

Thr	158	P	N	-	CK2	N	LP	-	E

Ser	169	Y	N	PKC	CAM2, CKI, IKK	N	LP	<1	E

Thr	176	N	Y	PKC	PKC	N	LP	-	E

Thr	184	Y	N	PKC	PKC	N	LP	-	E

Ser	203	P	Y	-	CKI, CDC2, IKK	N	VHP	-	E

Ser	204	Y	Y	PKC, ATM, DNAPK	ATM	Y	HP	>1	B

Ser	221	P	Y	GSK3	PKG, IKK	N	LP	<1	B

Ser	225	Y	Y	GSK3	CDC2, CDK, ATM	N	LP	>1	B

Ser	231	P	Y	-	CDC2, CDK	Y	VHP	-	E

Ser	232	N	Y	CDK5	IKK, PKB	Y	NHP	<1	B

Ser	233	P	Y	CDC2	IKK	Y	HP	<1	E

Thr	236	N	Y	CDC2	-	Y	VHP	-	E

Ser	238	N	Y	-	-	Y	-	-	E

Ser	239	N	Y	CDC2	-	Y	HP	-	E

Y = yes (threshold >0.5), P = probable (threshold > 0.1~0.5), N = No (threshold <0.1), VHP = very high potential (threshold ≥1.0), HP = high potential (threshold >0.8 <1.0), LP = low potential (threshold <0.8), <1 = low surface accessibility, >1 = high surface accessibility, 1 = low potential for solvent accessibility, 2 = high potential for solvent accessibility, B = Buried surface, E = Exposed surface,

### Prediction of O-linked glycosylation sites

The *O*-GlcNAc modification is known to be dynamic and analogous to phosphorylation. The prediction results obtained from YinOYang 1.2 for *O*-*β*-GlcNAc showed that there are many high potential *O*-*β*-GlcNAc sites for *O*-linked modifications. The human IGFBP-6 has 12 potential sites for *O*-*β*-GlcNAc modifications at Ser 144, 148, 152, 204, 231, 232, 233, 238 and 239 and at Thr 143, 146 and 236, with five Yin-Yang sites marked by asterisk (Figure 2).

OGPET also predict possible O-GlcNAc Ser and Thr sites. These are given in Table 2. These sites were almost same as predicted by YinOYang server.

### Identification of False-Negative Sites

There were 5 Yin Yang sites according to the prediction results (Ser: 144, 148, 152, 204 and Thr 146). Besides these, there were many other Ser and Thr residues that were predicted to be non-glycosylated, but the phosphorylation potential predicted was either much higher than the threshold value or very close to it. These are also conserved residues in mammals. Such residues appear to be as false-negative sites. These may act as possible Yin Yang sites other than those which have been predicted by the YinOYang 1.2 method. According to our results these sites may be Ser 120 and 169; and Thr 75 and 126 (Table 3).

**Table 3:** Proposed Ser/Thr residues for interplay of phosphorylation and *O*-GlcNAc modification in *Homo sapiens *IGFBP-6

SUBSTRATE		Predicted Yin Yang sites	Proposed FN-Yin Yang sites	Proposed Yin Yang sites	Yin Yang sites by similarity
**IGFBP-6**	Ser	144, 148, 152, 204	120, 169	204	-
	
	Thr	146	75, 126		-

## Discussion

Many studies have tried to find out the role of host factors in causing hepataocellular carcinoma linked to HBV and HCV. One of the factors mediating the HCV and HBV associated hepatocellular carcinoma is IGFs. HBV stimulate the expression of IGF-IR and modulate the transcription of IGFII using P3 and P4 promoter [7]. Whereas, in HCV associated hepatocellular carcinoma IGFII expression is activated by fetal promoters. HCV core protein has been found to increase the expression of IGFII through PKC pathway and plays role in HCV pathogenesis in inducing hepatocellular carcinoma [46].

Post-translational modifications in proteins play very important role in regulation of their functions and induce conformational changes that allow the protein to interact with other proteins [47,48]. Binding of IGFBP-6 with IGF-II inhibits IGF-II binding with cell surfaces (receptors) and decrease its affinity for proteolysis. Proteolysis of IGFBPs decreases their binding affinities with IGFs [20] and *O*-glycosylation of IGFBP-6 enhances its resistance to proteolysis by chymotrypsin and trypsin [19]. The IGFBP interaction with IGF can change from a high affinity stable complex to a highly labile one when IGF release is required. In the tissues, the release of IGFs from the IGFBPs can be modulated by three mechanisms; which function to decrease the affinity of the IGFBPs to the IGFs and act as a sustaining local source of IGFs to the IGF receptors. The first mechanism is association of the IGFBPs to the extracellular matrix (ECM) or specific cell membranes, second is the cleavage of the IGFBPs by specific proteases, and third is dephosphorylation [49].

**Figure 3:**
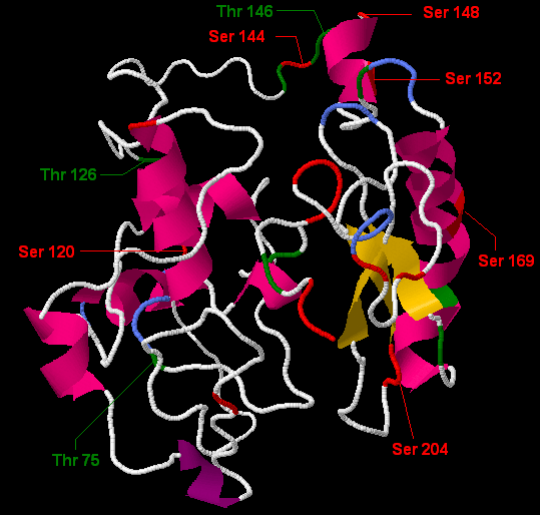
**A homology model of human IGFBP-6 utilizing automated protein modeling option was retrieved through I-TASSER server**. Five models were received from the server utilizing five different templates namely: model 1-5 through this option. Among the five, one that covered all amino acids with alpha helix structure and beta pleated sheet, high resemblance with experimentally determined C-terminal and high C-value was selected. This model shows that predicted Yin Yang sites have high surface accessibility for the phosphorylation and *O*-*β*-GlcNAc interplay. The Ser and Thr residues are denoted by red and green colors respectively.

Ser residues in the mid region of the IGFBP-6 are the main target of the phosphorylation. Headey et al (2004) found that the IGF-II binding site is located on C-terminal of IGFBP-6 from Leu 174, Gly 206 [21]. Regulation of IGF bioavailability, binding of IGFs to IGFBPs is modulated by phosphorylation process [22]. Phosphorylation of human IGFBPs enhances both its affinity for IGFs and their capacity to inhibit IGFs actions [50]. A phosphorylation site of IGFBP-6 at Thr-126 is described by Baxter and Firth which is phosphorylated by enzyme PKC^c ^and it is also a Yin Yang site [51]. Our Netphos 1.0 and Disphos results showed that IGFBP-6 has high potential for phosphorylation at middle and C-terminal region. As C-terminal of IGFBP-6 is involved as binding site for IGF-II, we found that Ser 204 has high potential for phosphorylation. It was also a conserved residue. Our results of NetPhos K 1.0 and Kinasephos for the prediction of phosphorylation potential of all Ser and Thr residues showed that these residues are phosphorylated by different kinases during cell cycle as shown in Table 2. These experimentally verified residues are conserved in IGFBP-6 and we can assume that these phosphorylated sites may be present on IGFBP-6 of other mammals "by similarity" where these phosphorylation sites are not yet experimentally known. *O*-*β*-GlcNAc modification can occur on these Ser and Thr residues where kinases are involved in phosphorylation as it is well known that kinases and OGT can compete for same site modification [34-37,52]. This shows a possibility for interplay between phosphorylation and OGT on these residues. *O*-glycosylation is known and experimentally verified post-translational modification in IGFBP-6 [53]. YinOYang 1.2 and OGPET prediction results had shown that IGFBP-6 have high potential for *O-*linked glycosylation (Figure 3).

The Ser and Thr residues of IGFBP-6 which are predicted to be phosphorylated and also showed positive potential for *O*-*β*-GlcNAc modification are Ser-144, 148, 152 and 204; and Thr 146. NetPhos 2.0 and Disphos prediction results showed that there are many Ser and Thr residues which are not predicted as Yin Yang but have high potential for phosphorylation, same as; YinOYang 1.2 and OGPET also predicted such type of residues to have high potential for *O*-*β*-GlcNAc modification (Table 1). These predicted sites can also be phosphorylated by different kinases (Table 2) and act as possible Yin Yang sites for *O*-*β*-GlcNAc modification (Table 3). These remaining Ser and Thr residues of IGFBP-6 which are conserved in different species and either known or predicted to be phosphorylated, showed negative potential for *O*-*β*-GlcNAc modification but very close to threshold value are known as false-negative Yin Yang (FN-Yin Yang) sites (Table 3). These conserved sites can be accessed by different kinases so that these sites have also strong possibility for OGT access and thus can also act as source of interplay for phosphorylation and *O*-*β*-GlcNAc [34-37]. In our study, Ser 120 and 169, and Thr 75 and 126 are predicted as FN-Yin Yang sites.

IGFBP-6 regulates biological processes such as cell proliferation or growth arrest [54]. Overexpression of IGFBP-6 in cancer cells activates programmed cell death [55]. *O-*glycosylation at mid region leads to free IGFBP-6 state by inhibiting binding to glycosaminoglycans and by protecting against proteolysis. Both glycosaminoglycan binding and proteolysis of IGFBP-6 decreases its binding affinity for IGF-II. As we know that C- domains of IGFBP-6 contains the binding sites and bind with IGF-II receptor with high affinity. Post translational modifications in the binding sites of IGFBP-6 change its affinity with IGF-II and glycosaminoglycans [21]. The mutations in C-terminal domain of IGFBP-6 result in reduction for IGF-II binding affinity. The Ser 203, Ser 204 and Gln 205 contribute to the IGF-II binding preference of IGFBP-6. The proximity of the IGF-II and glycosaminoglycan binding sites provides a structural basis for the decrease in IGF binding affinity after IGFBP-6 interaction with glycosaminoglycans. IGFBP-6 also has putative PKC site in C-terminal [22]. Ser 204 is conserved residue in mammals. Our results indicate that Ser 204 has high potential for phosphorylation and it can be phosphorylated by PKC. Ser 204 is also a Yin Yang site and has high potential for *O-*glycosylation. The sites needed for IGF-II binding of IGFBP-6 are present in C-terminal. In the binding sites at C-terminal of IGFBP-6 there are two Ser residues 203 and 204. Our results showed that only Ser 204 residue is conserved and has ability to be phosphorylated and glycosylated.

To verify possible Yin Yang sites, we sketched the 3D structure of IGFBP-6 protein (Figure 3). We evaluated the surface accessibility of IGFBP-6 for these post translational modifications (Table 2). We found that Ser 204 was predicted as "Buried" by Netsurf P server, while scan site showed high accessibility for solvents [56]. This problem was resolved by assessing the experimentally determined 3D structure of C-terminal deposited by Headey et al (2004). We were amazed to see that our predicted full length IGFBP-6 3D structure having the same C-terminal as experimentally proved by Heedy et al. (2004), and it clearly showed that Ser 204 of IGFBP-6 is accessible for these types of modifications (Figure 3).

**Figure 4:**
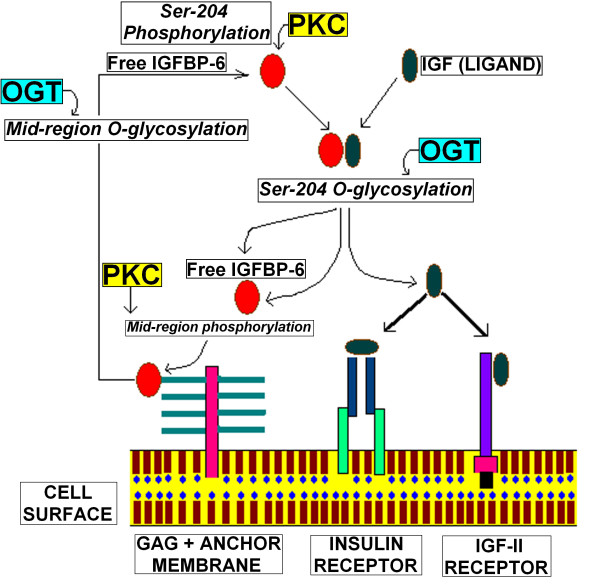
**Schematic diagram illustrating the role of IGFBP-6 phosphorylation and *O*-glycosylation on IGF-II functions**. Here we propose that alternative *O*-*β*-GlcNAc modification and phosphorylation of Ser 204 control the binding of IGF-II with IGFBP-6 during viral infection, while mid region phosphorylation and *O*-*β*-GlcNAc modifications controls it's binding with glycosaminoglycans.

As we know that there is competition between *O-*glycosylation and phosphorylation on Ser and Thr residues [57], we therefore, propose that *O*-*β*-GlcNAc and phosphate modifications at Ser 204 residue control the binding of IGFBP-6 with IGF-II, while *O*-glycosylation and phosphorylation on middle region Ser and Thr residues control the binding of IGFBP-6 with glycosaminoglycans. Since IGFBP-6 has shown an inhibitory effect on IGF functions in many cancer cell lines for example in human breast cancer, so reduced IGFBP-6 levels can therefore, affect cell growth in multiple ways, such as increasing IGF bioavailability and reducing IGF independent growth inhibitory effects etc. So we can conclude that due to *O*-*β*-GlcNAc modification at Ser 204, binding of IGFBP-6 with IGF-II reduced and resulting in binding of IGF-II with IGF-II receptor and promote cancer progression which can lead to hepatocellular carcinoma in HCV infected patients (Figure 4). The application of such predictive techniques may be of interest to develop therapeutics to decrease the hepatocellular carcinoma in cases with HCV and HBV-related chronic hepatitis.

## Abbreviations

HCV: Hepatitis C virus; IGF: insulin growth factor; IGFBPs: insulin like growth factor binding proteins

## Competing interests

The authors declare that they have no competing interests.

## Authors' contributions

AW and SK contributed equally to this study. AW, SK and HS designed the study. IB, AS, GS, KH, NN, NS, FK, SMT and SI analyze the data and wrote paper. AW, IB, SK, IB and HS approve finalized version of paper. All work was performed under supervision of HS. All authors read and approved the final manuscript.

## Authors' information

Bushra Ijaz (M Phil Molecular Biology), Waqar Ahmad (M Phil Chemistry) and Gull S (MSc Biochemistry) are Research Officer; Shabbiri K is lecturer while Nazar S, Nazar N and Fouzia K are BS (Hons) student at GC University, Lahore. Kausar H, Sawar MT, Shahid I and Asad S are PhD scholars. Sajida Hassan (PhD Molecular Biology) is Principal Investigator at CEMB, University of the Punjab, Lahore
